# Human Rhinovirus 3C protease cleaves RIPK1, concurrent with caspase 8 activation

**DOI:** 10.1038/s41598-018-19839-4

**Published:** 2018-01-25

**Authors:** Sarah N. Croft, Erin J. Walker, Reena Ghildyal

**Affiliations:** Centre for Research in Therapeutic Solutions, Health Research Institute, University of Canberra, Canberra, ACT Australia

## Abstract

Human Rhinovirus (HRV) is a pathogen of significant medical importance, being a major cause of upper respiratory tract infections (common colds) as well as causing the majority of virus-induced asthma exacerbations. We investigated whether HRV could modulate apoptosis, an innate antiviral response. Apoptotic signals are generated either extrinsically or intrinsically and are propagated via caspase cascades that lead to cell death, reducing viral replication, which relies on cellular machinery. Using HRV16 infected cells, in combination with chemical inducers and inhibitors of extrinsic apoptosis we show that HRV16 3C protease cleaves a key intermediate in extrinsic apoptosis. Receptor-interacting protein kinase-1 (RIPK1), an extrinsic apoptosis adaptor protein, was cleaved by caspase 8, as expected, during chemical induction of apoptosis. RIPK1 was cleaved in HRV infection albeit at a different site. Caspase 8 activation, which is associated with extrinsic apoptosis, was concurrent with HRV 3C protease mediated cleavage of RIPK1, and potentially increased the accessibility of the HRV 3C cleavage site within RIPK1 *in-vitro*. The caspase 8 mediated RIPK1 cleavage product has a pro-apoptotic function, and further cleavage of this pro-apoptotic cleavage product by HRV 3C may provide a mechanism by which HRV limits apoptosis.

## Introduction

Human Rhinovirus (HRV) is a small virus with a single stranded, positive sense RNA genome that belongs to the *Picornaviridae* family^1,2^. HRV is the main viral causative agent of the common cold, and the major viral cause of asthma exacerbations^3,4^. The HRV genome encodes structural and functional proteins, of which three proteases (2A, 3CD and 3C) enable viral polyprotein maturation^5^ and have been shown to cleave host proteins. The cleavage of translation machinery^5^, transcription factors^6^ and nucleoporins^7,8^ contributes to host cell shutoff, leading to upregulation of viral translation and transcription. In response to viral infection, the host cell can undergo apoptosis, a method of controlled cell suicide that does not provoke an inflammatory response^9,10^. As viruses rely on host cellular proteins such as translation machinery for replication, initiating cell death would ultimately inhibit viral replication and disrupt the infection cycle^11^. HRV, like other *Picornaviridae*, is thought to be capable of regulating the apoptotic process through the activity of its proteases towards cellular apoptotic factors^12,13^. The delay or inhibition of apoptosis would allow optimal viral replication and subsequently optimal release and infection.

Extrinsic apoptosis is induced within the cell in response to external harmful stimuli and is dependent on the activation of caspase 8^14,15^. Caspase 8 is recruited to the cytoplasmic portion of death receptors that have formed complexes with other death inducing adaptor proteins such as Fas-Associated protein with Death Domain (FADD), Tumor necrosis factor receptor type 1-associated Death Domain protein (TRADD)^14,16^ and Receptor-interacting protein kinase-1 (RIPK1)^15,17^.

RIPK1 is cleaved by caspase 8 resulting in a c-terminal fragment that functions in a pro-apoptotic manner by strengthening the association and interactions of the death receptor complex (15, 17, 18). Importantly, RIPK1 is central to the host apoptotic response to HRV infection^18^. Retinoic-acid Inducible Gene-I (RIG-I) and Melanoma Differentiation-Associated protein-5 (MDA-5) are cytoplasmic viral RNA sensors that, once activated, bind Interferon-β Promoter stimulator-1 (IPS-1)^19,20^ followed by recruitment of the proapoptotic proteins FADD and caspase 8. Caspase 8 is then activated through the formation of a caspase 8/RIPK1/FADD/IPS-1 complex, resulting in induction of the extrinsic apoptosis pathway^18,21,22^. Additionally, the binding of dsRNA to Toll-like receptor (TLR) 3 results in the recruitment of TIR-domain-containing adapter-inducing interferon-ß (TRIF) to TLR 3^23,24^. RIPK1 is bound to TRIF when complexed with TLR 3, through the RIP homotypic interaction motif (RHIM) domain within RIPK1 and results in caspase 8 activation through the c-terminal Death Domain of RIPK1^23–25^.

While it is known that HRV proteases can alter cellular factors, little is known about HRV induced changes to apoptotic signalling processes. In the present study, we have investigated the cleavage of RIPK1 in HRV infection. RIPK1 was cleaved in cells infected with HRV16, generating a RIPK1 cleavage product that was different from that seen with caspase 8 cleavage. The addition of exogenous HRV 3C protease produced the same RIPK1 cleavage product, strongly suggesting that 3C protease is responsible for the observed HRV16 specific cleavage of RIPK1. Additionally, inhibition of HRV 3C activity abolished viral RIPK1 cleavage. Interestingly, data from *in vitro* protease assays strongly suggest that cleavage of RIPK1 by 3C protease can occur downstream of caspase 8 mediated RIPK1 cleavage. As caspase 8 mediated cleavage of RIPK1 is an early apoptotic event^26^, HRV may be able to limit the progression of apoptosis to its effector phase through cleavage of the pro-apoptotic, caspase 8 generated RIPK1 cleavage product.

## Results

### RIPK1 is cleaved by caspase 8 in the apoptotic cascade

**Figure 1:**
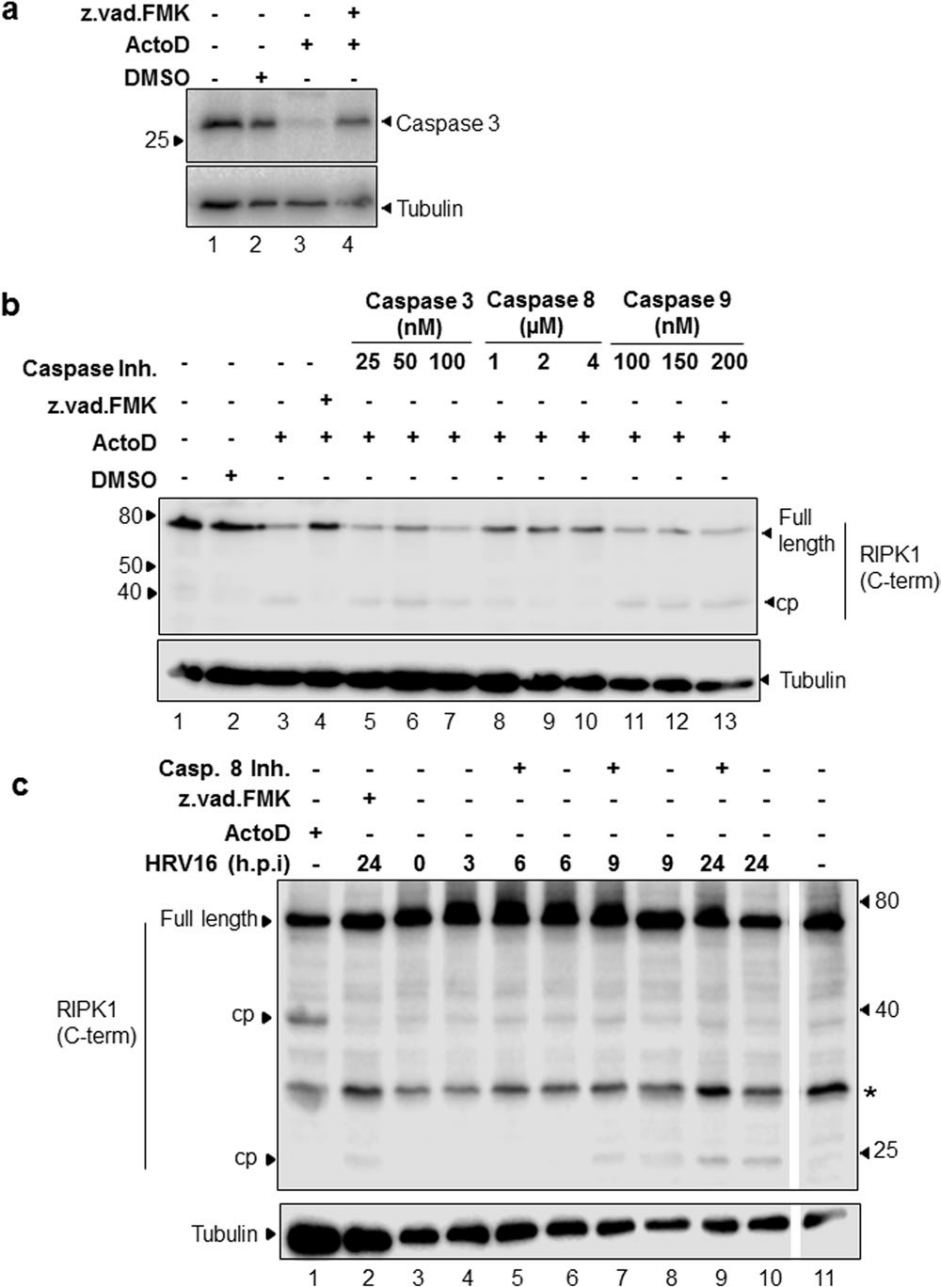
ActoD induced apoptosis leads to caspase 3 activation and caspase 8-dependent RIPK1 cleavage, different from that seen in HRV16 infection. O-HeLa cells were either treated with ActoD at 5 µg/mL or left untreated. One hour post treatment, samples were treated with DMSO or indicated caspase inhibitors for 16 hours. Cells were then lysed and proteins collected for western blot analysis. (**a**) Lysates of cells that were left untreated, treated with DMSO alone, ActoD alone or ActoD followed by z.vad.fmk were electrophoresed and proteins transferred to nitrocellulose membrane. Non-specific sites on the membranes were blocked with 4% skim milk followed by probing with anti-caspase 3 antibody (upper blot) or anti-tubulin antibody (lower blot) as loading control. The position of bands correlating with full length caspase 3 or tubulin is indicated on the right and relevant molecular weight markers (in kDa) on the left. (**b**) Lysates of cells that were left untreated, treated with DMSO alone, ActoD alone, ActoD followed by z.vad.fmk or specific caspase inhibitors were electrophoresed and proteins transferred to nitrocellulose membrane. After blocking non-specific sites with 4% skim milk, membranes were probed with anti-RIPK1 antibody (upper blot) or anti-tubulin antibody (lower blot) as loading control. Concentrations of the specific caspase inhibitors are indicated. The position of bands correlating with full length RIPK1 or tubulin is indicated on the right and relevant molecular weight markers on the left. Cropped, relevant sections of the blots are shown for clarity, with the full length blot included in Supplementary Information, Figures S2 and S3. (**c**) O-HeLa cells were infected with HRV16 at M.O.I of 3. Cells were treated with caspase 8 inhibitor (4 µM) or z.vad.FMK (20 µM) at 4 h.p.i or left untreated. An uninfected sample was treated with ActoD (5 µg/mL) for 9 hours. Cells were lysed at indicated times (h. p. i.) and proteins collected for western blot analysis. Membranes were probed with anti-RIPK1 (upper blot), or anti-tubulin antibodies (lower blot) as loading control. Full length RIPK1 and its cleavage products (cp) are indicated on the left and relevant molecular weight markers on the right. *nonspecific band at ~30 kDa.

Treatment of O-HeLa cells with ActoD resulted in induction of apoptosis as evidenced by the cleavage of full length caspase 3 (Fig. 1a), as expected^27^; this was reversed with treatment with the pan-caspase inhibitor z.vad.FMK (Fig. 1a, compare lane 3 and 4). RIPK1 was cleaved in ActoD treated cells as evidenced by the loss of full length RIPK1 and appearance of a ~38 kDa band (Fig. 1b, compare lane 3 with lanes 1, 2). RIPK1 cleavage was caspase dependent as addition of z.vad.FMK to ActoD treated cells resulted in abrogation of cleavage (Fig. 1b, compare lane 4 with lane 3). Treatment of cells with caspase 8 inhibitor following ActoD treatment resulted in a dose dependent reduction in RIPK1 cleavage (Fig. 1b, lanes 8–10); neither caspase 3 nor caspase 9 inhibitors had any effect (Fig. 1b, lanes 5–7, 11–13). Our data suggest that RIPK1 cleavage in ActoD induced apoptosis is dependent on caspase 8.

### RIPK1 is cleaved in HRV16 infected cells

The effect of HRV16 infection on RIPK1 cleavage was investigated. Cells were infected with HRV16, treated with either z.vad.FMK or specific caspase 8 inhibitor at 4 h.p.i. or left untreated, and lysed at indicated time-points (Fig. 1c). Uninfected cells treated with ActoD or left untreated served as additional controls. Cell lysates were analysed for RIPK1, with tubulin included as a loading control. As expected, a ~38 kDa RIPK1 caspase 8 cleavage product was observed in ActoD treated cells (lane 1). A 23 kDa RIPK1 cleavage product was observed in HRV16 infected cell lysates after 9 h.p.i. There was no observable change in 23 kDa cleavage product in presence of caspase 8 inhibitor compared to corresponding untreated lysates (compare lanes 7,8 and 9,10). The cleavage of RIPK1 in HRV16 infected cells was not prevented when caspases were inhibited with z.vad.FMK (compare lanes 2,10). Neither the ~38 kDa nor 23 kDa RIPK1 cleavage products were detected in untreated, uninfected lysate (lane 11). A non-specific band of approximately ~30 kDa was present in all samples and is currently uncharacterised. Our data show that RIPK1 is cleaved in HRV16 infection in a caspase independent manner.

### Inhibition of 3C protease prevents cleavage of RIPK1 in HRV16 infected cells

**Figure 2:**
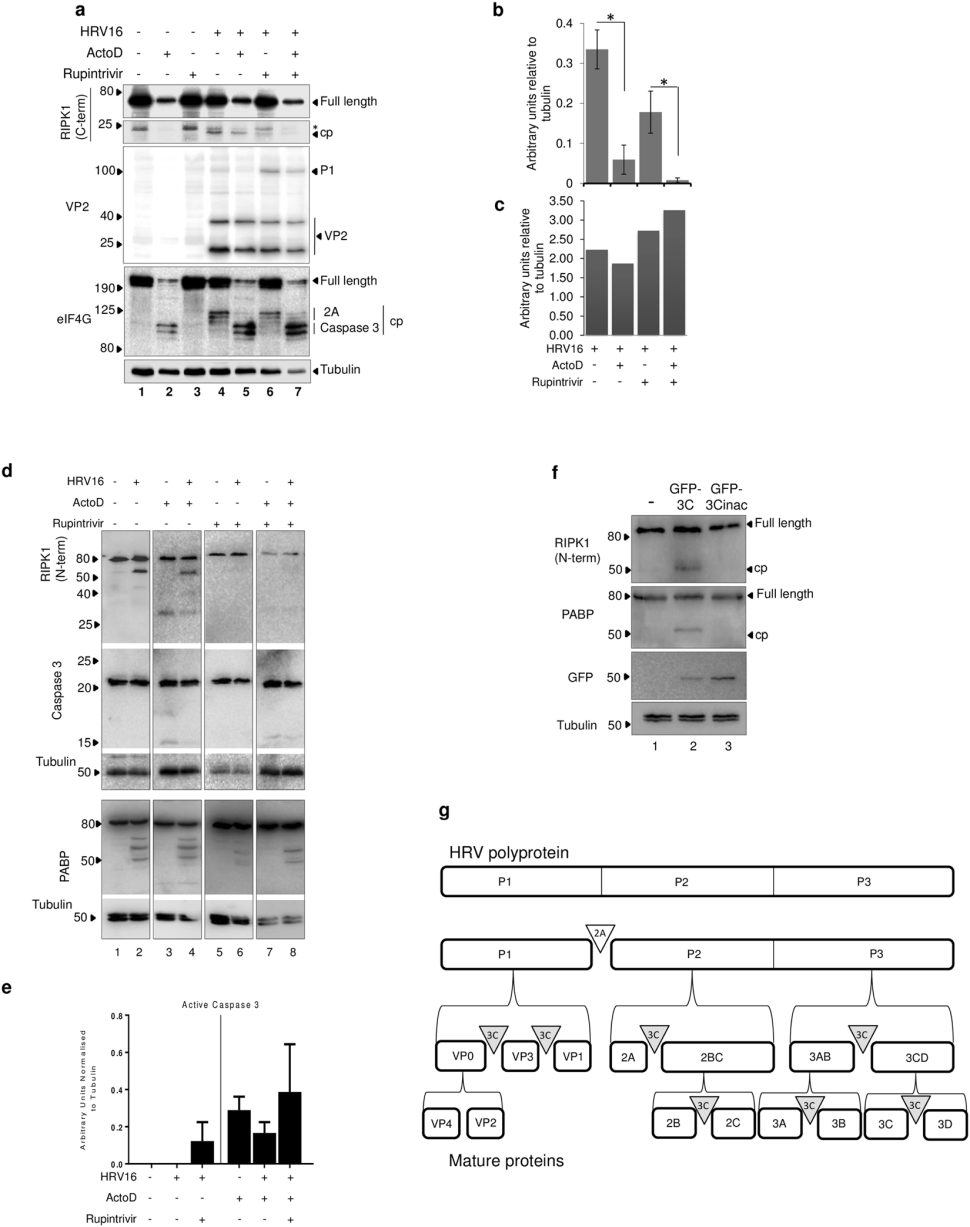
HRV16 induced cleavage of RIPK1 is inhibited with treatment with Rupintrivir. O-HeLa cells were infected with HRV16 at an M.O.I of 3 or left uninfected, followed by treatment with ActoD (5 µg/mL) at 3 h.p.i. and/or Rupintrivir (1 µM) at 9 h.p.i or left untreated. Cells were then lysed and proteins collected for western blot analysis. (**a**) Membrane was probed with anti-RIPK1 (c-terminal) antibody, anti-VP2 antibody, anti-eIF4G antibody or anti-tubulin antibody as loading control. Representative blots from one experiment are shown; antibodies used are indicated on the left, bands specific for P1, VP2, eIF4G, RIPK1 and tubulin are indicated on the right, and relevant molecular weight markers to the left of the blot. *Denotes non-specific bands; cp – cleavage product. (**b**) Band intensities of the 23 kDa viral RIPK1 cleavage product detected in a) were quantitated using ImageJ and normalised to tubulin. GraphPad Prism was used to ascertain statistically significant differences between samples treated with Rupintrivir as compared to respective infected samples. *p < 0.05. Data are mean +/− standard error of the mean of three independent experiments. (**c**) Band intensity representative of P1 detected in a) was quantitated using ImageJ. Results were normalised to tubulin and expressed as arbitrary units normalised to tubulin. Data are mean +/− standard error of the mean of three independent experiments. (**d**) Membrane was probed with anti-RIPK1 (n-terminal) antibody, anti-caspase 3 antibody, anti-PABP antibody or anti-tubulin antibody as loading control. Representative blots from one experiment are shown; antibodies used are indicated on the left, bands specific for caspase 3, active caspase 3, PABP, RIPK1 and tubulin are indicated on the right, and relevant molecular weight markers to the left of the blot. (**e**) Band intensity representative of active caspase 3 (17 kDa) detected in d) was quantitated using ImageJ. Results were normalised to tubulin and expressed as arbitrary units normalised to tubulin. Data are mean +/− standard error of the mean of three independent experiments. (**f**) GFP-3C (active) or GFP-3Cinac (inactive) constructs were transfected into a sub-confluent monolayer of A549 cells. At 16 hours post-transfection cells were lysed and proteins collected. Cell lysates were then processed for western blot analysis. Membrane was probed with primary anti-RIPK1 (n-terminal) antibody, with anti-tubulin antibody included as a loading control. Primary anti-PABP antibodies were included to validate activity or inhibition of viral proteases. Anti-GFP antibodies were included to confirm transfection. Proteins were detected with ECL. Molecular weight markers are to the left of the blot. The image is representative of three independent experiments. Cropped, relevant sections of the blots are shown for clarity, with the full length blot included in Supplementary Information, Figures S4 and S5. (**g**) Schematic diagram depicting HRV polyprotein processing into mature proteins. Translation of RNA genome into a large single polyprotein is followed by the 2A mediated cleavage (unfilled triangle) between P1 and P2 protein regions. Subsequent cleavages are mediated by the 3C protease (grey triangles) or by autocatalysis (no triangle).

To determine if RIPK1 is cleaved by HRV16 3C, uninfected or infected O-HeLa cells were treated with ActoD to induce apoptosis at 3 h.p.i., alone or in combination with the HRV 3C protease inhibitor, Rupintrivir added at 9 h.p.i. Cell lysates were collected at 16 h.p.i, followed by Western blot analysis for RIPK1. As expected, a 23 kDa RIPK1 cleavage product was observed in samples subject to infection, but without treatment of Rupintrivir, correlating with a decrease in full length RIPK1 (Fig. 2a, lanes 4,5 in RIPK1 blots). Significantly less 23 kDa RIPK1 cleavage product was observed in samples treated with Rupintrivir as compared to infected samples without (compare lanes 4 and 6) or with (compare lanes 5 and 7) ActoD. This was confirmed by densitometric analysis of RIPK1 specific bands normalised to tubulin (p = 0.01 and p = 0.03 respectively, Fig. 2b).

Activity of HRV16 3C protease was clearly inhibited by Rupintrivir treatment as viral polyprotein (P1) processing was downregulated; 3C protease is responsible for the cleavage of P1 to derive virus capsid proteins (VP1-4) (Fig. 2g)^28^. Rupintrivir treatment resulted in increased level of P1 (Fig. 2a, lanes 6,7 in VP2 blot) compared to infected cells not treated with Rupintrivir, with or without ActoD, correlating with decrease in level of 23 kDa RIPK1 product (lanes 6,7 in RIPK1 blot). Additional experiments showed the presence of an ~50 kDa, n-terminal RIPK1 cleavage product in samples subject to infection with HRV16 (Fig. 2d lanes 2 and 4) but not those subject to infection and treatment with Rupintrivir (Fig. 2d Lanes 6 and 8). The cleavage of RIPK1 paralleled cleavage of a known 3C protease substrate Poly-A-Binding protein (PABP), which was used to confirm 3C protease activity. Densitometric analysis of P1 specific band, normalised to tubulin showed an increase in the presence of Rupintrivir, compared to HRV16 infection alone (Fig. 2c), confirming the inhibition of 3C protease activity. HRV 2A protease activity was confirmed in all infection samples, as evidenced by the virus specific cleavage of eIF4G (Fig. 2a, lanes 4–7, eIF4G blot) which did not parallel RIPK1 cleavage, eliminating 2A as the protease responsible for RIPK1 cleavage. The observed 2A protease activity suggests that any decrease in cleavage of RIPK1 after Rupintrivir treatment was not due to an overall decrease in viral protease activity. Additionally, no significant decrease in total viral protein synthesis was observed in samples treated with Rupintrivir as compared to corresponding untreated samples (Fig. 2a).

The ~23 kDa c-terminal RIPK1 cleavage product was not directly due to induction of extrinsic apoptosis as it was not observed in samples treated with ActoD alone (Fig. 2a, lane 2, RIPK1 blots) and was not increased in infected samples treated with ActoD with or without Rupintrivir (compare lanes 5 and 7 with lanes 4 and 6 respectively, RIPK1 blots). ActoD treatment resulted in loss of full length RIPK1 and induced apoptosis as was confirmed with the caspase 3 mediated degradation of eIF4G as previously described^29^ (lanes 2,5,7, eIF4G blot).

Amounts of active caspase 3 (17 kDa) were examined to assess any induced apoptosis in samples subject to infection in combination with ActoD or Rupintrivir treatment. As shown in Fig. 2d and e, there is increased caspase 3 activation in HRV16 infected samples treated with Rupintrivir as compared to infection only samples. Additionally, amounts of active caspase 3 increased with ActoD treatment as expected and increased further with ActoD and Rupintrivir treatment.

### HRV16 3C protease cleaves RIPK1 in lung alveolar cells

To determine whether the HRV16 3C protease was able to cleave RIPK1 in lung cells, sub-confluent layers of A549 cells were transfected with GFP-tagged active 3C protease (GFP-3C) or an inactive mutant of 3C (GFP-3Cinac). Cell lysates were collected at 16 hours post transfection, followed by western blot analysis. Cleavage of a known target of 3C, PABP, was used to confirm activity of the 3C protease. As expected, a RIPK1 cleavage product was detected in the GFP-3C expressing sample, but not in the GFP-3Cinac or untransfected samples (Fig. 2f, compare lane 1 with lanes 2, 3 in RIPK1 blot). GFP-3Cinac was expressed to higher levels than GFP-3C (compare lanes 2 and 3 in the GFP blot), however, PABP was cleaved only in the GFP-3C transfected sample. Clearly, RIPK1 was cleaved in cells expressing GFP-3C but not -3Cinac.

Our data (Figs 1 and 2) strongly suggest that 3C protease cleaves RIPK1 in HRV16 infected cells to generate an ~23 kDa c-terminal and an ~50 kDa n-terminal product; that 2A protease is not involved in this cleavage and that this cleavage is not due to cellular apoptotic response. Importantly, 3C protease cleavage of RIPK1 is observed in a clinically relevant alveolar cell line.

### Prior cleavage of RIPK1 by caspase 8 does not abrogate 3C protease mediated cleavage *in-vitro*

**Figure 3:**
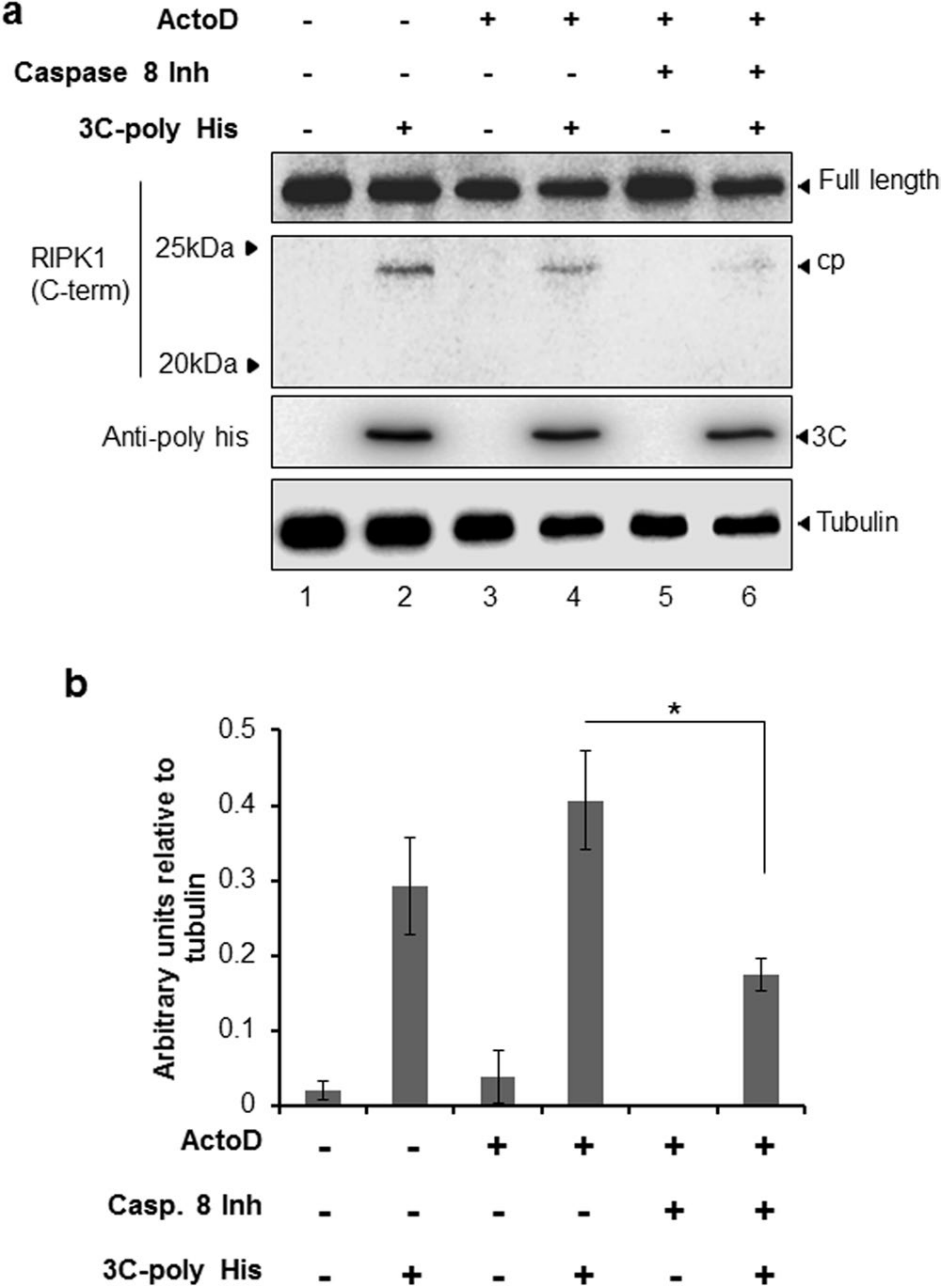
HRV 3C protease cleaves RIPK1 to produce a 23 kDa cleavage product. O-HeLa cells were either treated with ActoD and/or caspase 8 inhibitor (1 hour post ActoD treatment) or remained untreated as indicated. Cells were lysed 9 hours later and proteins collected. HRV 3C was added where indicated and samples were incubated for 6 hours before stopping the protease reaction. Cell lysates were then processed for western blot analysis. (**a**) Membrane was probed with anti-RIPK1 (c-terminal) antibody, anti-poly His antibody with anti-tubulin antibody included as loading control. (**b**) Band intensity representative of HRV 3C induced 23 kDa RIPK1 cleavage product detected in a), was quantitated using ImageJ. Results were normalised to tubulin and expressed as arbitrary units. Experiments were repeated three times and relative protein intensities are represented as mean +/− standard error of the mean. *p ≤ 0.05. Cropped, relevant sections of the blots are shown for clarity, with the full length blot included in Supplementary Information, Figure S6.

We used *in vitro* protease assays to investigate the relationship of extrinsic apoptosis with cleavage of RIPK1 by 3C protease. O-HeLa cells were treated with ActoD alone to induce apoptosis or with caspase 8 inhibitor and lysed in the absence of protease inhibitors. The lysates were incubated with HRV14 3C (poly His tagged) protease for 6 hours followed by Western blot analysis. A 23 kDa RIPK1 cleavage product was observed only in samples treated with 3C protease (Fig. 3a, lanes 2, 4, 6, RIPK1 blot), suggesting that 3C protease can cleave RIPK1 giving rise to the product observed in HRV16 infection. Induction of apoptosis by ActoD prior to 3C protease assay resulted in increased cleavage (increased 23 kDa cleavage product relative to tubulin, lane 4); this was reduced by caspase 8 inhibitor (lane 6). Quantitation of the 3C induced RIPK1 cleavage product supports these observations, with HRV16 3C dependent cleavage of RIPK1 up regulated in ActoD treated cells, as compared to untreated control and a significant reduction of cleavage in samples treated with ActoD and caspase 8 inhibitor prior to 3C protease assay (Fig. 3b; p = 0.007 ActoD + 3C vs ActoD + caspase 8 inhibitor + 3C).

The data in Fig. 3 confirms that RIPK1 cleavage in HRV infected cells is mediated by 3C protease. Additionally, induction of apoptosis did not abrogate this cleavage; indeed caspase 8 activity may facilitate the cleavage of RIPK1 by 3C protease.

### Apoptosis induction reduces viral replication

**Figure 4:**
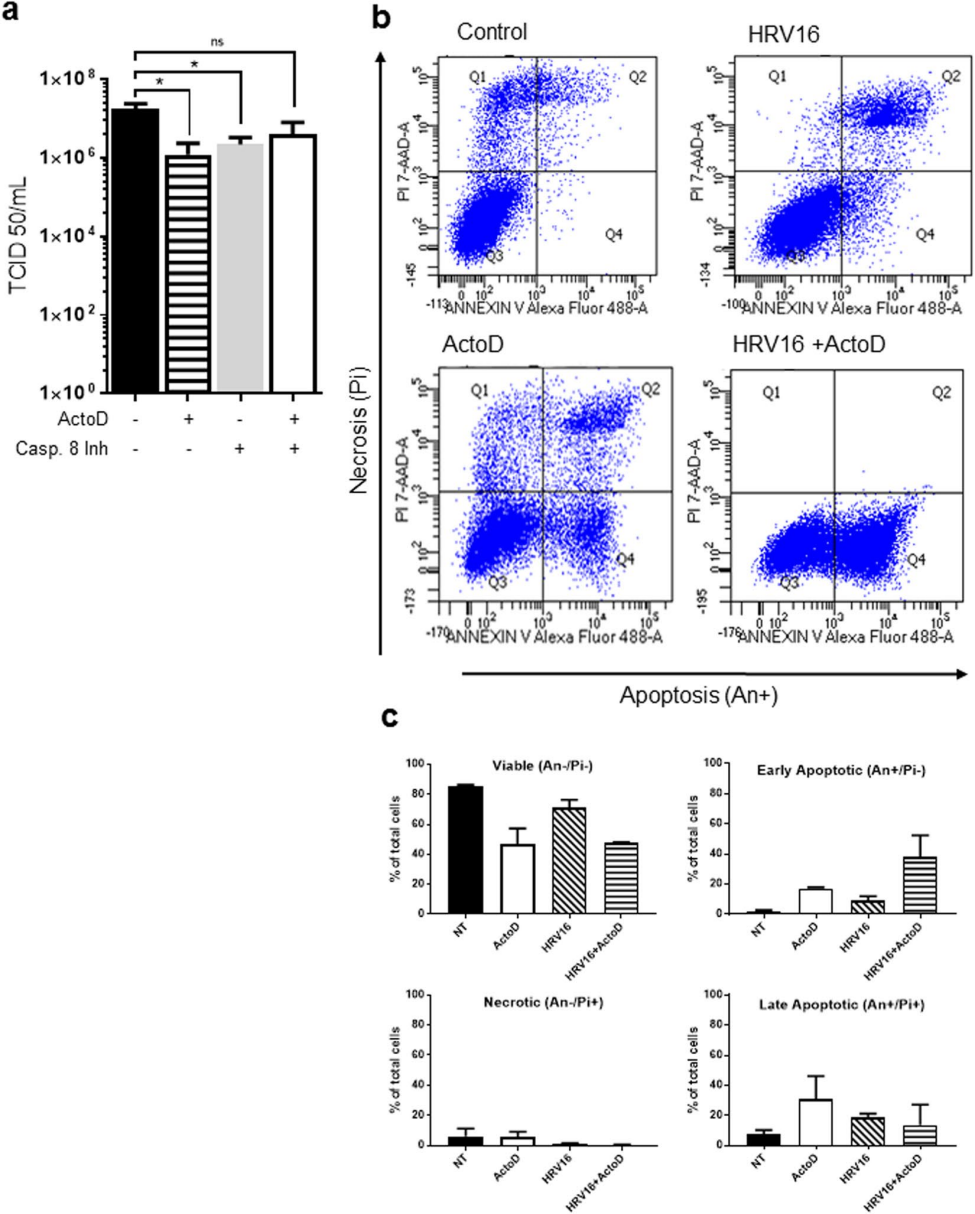
(**a**) HRV16 replication is down regulated with inhibition of caspase 8. O-HeLa cells were infected with HRV16 at an M.O.I of 3. At 3 h.p.i., ActoD (5 µg/mL) was added to selected samples. Caspase 8 was inhibited as indicated through addition caspase 8 inhibitor (4 µM) at 4 h.p.i. Cultures were frozen at 24 h.p.i. Once thawed, virus was clarified and titrated. Results are expressed as mean of 3 independent experiments. Error bars are +/− standard error of the mean. *p ≤ 0.05, ns – nonsignificant. (**b**) HRV16 restricts apoptotic pathways. O-HeLa cells were infected and treated with ActoD as in a) above. Cells were collected at 12 h.p.i and the effect of treatment and/or infection analysed by flow cytometry after staining with annexin V-FITC (An) and propidium iodide (Pi). Representative dual-parameter fluorescence density blots were acquired. (**c**) Bars are representative of percentage of viable (An−/Pi−), early apoptotic (An+/Pi−), late apoptotic (An+/Pi+) or necrotic (An−/Pi+) cells of total number of cells within each sample.

We next investigated whether inhibition of apoptosis, and specifically caspase 8, had an effect on viral replication. HRV16 infected cells were treated with ActoD with or without caspase 8 inhibitor 1 hour later, or with caspase 8 inhibitor alone and infectious virus titres measured at 24 h.p.i. Viral titres were significantly reduced by ActoD treatment (p = 0.0277) and caspase 8 inhibitor alone (p = 0.0387) but this reduction was not observed when caspase 8 activity was inhibited after ActoD treatment (Fig. 4a). However, viral titres were non-significantly increased in HRV16 infected cells treated with ActoD followed by caspase 8 inhibitor compared to HRV16 infected cells treated with ActoD alone.

### HRV16 infection alters ActoD induced cell death

O-HeLa cells were left untreated (control), infected with HRV16, treated with ActoD to induce apoptosis, or infected with HRV16, then treated at 3 h.p.i. Cells were harvested at 12 h.p.i. and subjected to Annexin V-FITC labelling (apoptosis) and Pi staining (necrosis) followed by flow cytometry. Control cells, as expected were mostly healthy (Fig. 4b,c, >80% negative for Annexin V or Pi), with a small population (<10%) showing signs of necrosis (positive for Pi) or late apoptosis (positive for both Annexin V and Pi). ActoD treatment alone resulted in increased apoptosis, as expected, with ~50% cells showing signs of apoptosis (positive for Annexin V only or both Annexin V and Pi); there were ~10% necrotic cells (positive for Pi only). Infection with HRV16 resulted in ~30% of cells in early or late apoptosis (positive for Annexin V only or both Annexin V and Pi) and negligible necrotic cells. ActoD treatment in the presence of HRV16 infection did not result in late apoptosis, with cells equally divided between healthy and early apoptotic phenotypes (Fig. 4b, compare cell scatter plot labelled ActoD with that labelled HRV16+ ActoD). The percentage of cells present in each quadrant for the control, ActoD treatment and ActoD+ HRV16 infection are shown in Fig. 4c. These results confirm that ActoD treatment in the presence of HRV16 infection causes reduced late apoptosis (An+/Pi+) and necrosis (An−/Pi+) with an increase in early apoptosis (An+/Pi−) compared to treatment with ActoD alone.

Our data in Fig. 4 show that apoptosis progression, as induced by continued treatment with ActoD, is detrimental to HRV16, and leads to reduction in infectious titres. However, in the presence of HRV16 infection, ActoD is restricted from causing the full effects of apoptosis, suggesting that HRV16 is able to contain apoptosis progression.

## Discussion

The data presented in the current study indicates that HRV16 modulates the induction of extrinsic apoptosis in infected cells by cleaving RIPK1, a key apoptosis intermediate induced in response to HRV infection, and that this cleavage is accomplished by 3C protease.

RIPK1 was cleaved in cells infected with HRV16, however, in contrast to the 38 kDa product produced by caspase 8 cleavage (Fig. 1b), a c-terminal 23 kDa cleavage product is produced during HRV16 infection (Figs 1c and 2a). Figure 2c shows an additional RIPK1 band at ~30 kDa, it is present in all samples and further work is required to elucidate its function/relevance. The addition of the 3C protease inhibitor Rupintrivir led to a reduction in the c-terminal 23 kDa cleavage product seen in infection (Fig. 1c), suggesting that HRV16 3C protease is responsible for the virus-induced RIPK1 cleavage product. It is unlikely that HRV16 2A protease is responsible for cleavage of RIPK1, as HRV16 2A protease activity was still observed in Rupintrivir treated samples (Fig. 2a, eIF4G blot), while 3C protease activity was decreased, as evidenced by a lack of viral polyprotein maturation (Fig. 2a, P1 band in the VP2 blot).

The *in vitro* addition of the HRV 3C protease to whole cell lysate resulted in the same c-terminal 23 kDa RIPK1 cleavage product (Fig. 3a), further confirming that HRV 3C is likely the viral protease responsible for cleavage of RIPK1 during HRV16 infection and this was confirmed to be the c-terminal of RIPK1 (Fig. S2). Additionally, transfection of a GFP-3C construct into lung alveolar cells resulted in the same viral-induced cleavage of RIPK1 as seen in previous experiments performed in O-HeLa cells (Fig. 2f), indicating this is not a cell line specific event.

Most viruses rely on the cellular processes of a viable cell for their replication and assembly and as such, induction of apoptosis disrupts viral replication and limits infection^30^. The initiation of extrinsic apoptosis can occur via a number of receptors including the dsRNA sensors RIG-I, TLR-3 and MDA-5, which upon activation lead to the formation of an apoptosis signalling death complex^10^. Caspase 8 is recruited to this complex and is autophosphorylated, inducing autocleavage and activation^31^. The activation of caspase 8 has immediate effects such as the cleavage of RIPK1^15^. RIPK1 is a serine-threonine kinase that in its full length form promotes a pro-inflammatory response to specific cytokines^15^. Full length RIPK1 sustains a pro-survival or pro-inflammatory signal through NF-kB activation and interferes with FADD and TRADD interactions^15^. The caspase 8 dependent cleavage of RIPK1 during apoptosis inhibits the pro-survival/inflammatory effects, and the generated c-terminal 38 kDa cleavage product enhances interactions between death receptor-associated adaptor proteins FADD/TRADD, thus sensitising the cell to extrinsically induced apoptosis^15^. Using *in vitro* protease assays, we show that initial induction of extrinsic apoptosis may promote 3C protease cleavage of RIPK1 (Fig. 3b), as cell lysates treated with ActoD alone showed an increase in RIPK1 cleavage compared to untreated and caspase 8 inhibitor treated lysates. The 3C mediated cleavage of RIPK1 in lysates treated with ActoD was also increased, albeit non-significantly, as compared to untreated lysates. These results suggest that caspase 8 cleavage of RIPK1 may induce a conformational change in RIPK1, subsequently allowing greater accessibility to the HRV 3C cleavage site. The upregulation of viral cleavage of RIPK1 with caspase 8 activation was not seen in the infection models (Fig. 2b). This difference may be due to an increased accessibility of RIPK1 during the cell lysis process, however the prior cleavage of RIPK1 by caspase 8 did not abolish HRV cleavage, indicating that the two cleavage events do not negate each other.

As the caspase 8 inhibitor alone reduced viral titre (Fig. 4a) it could be proposed that some caspase 8 activation is beneficial to viral replication. However, the down regulation of viral replication with induction of apoptosis is widely reported^11,32^, and as expected^26^, prolonged activation of caspase 8 (ActoD treatment alone) commits the cell to the apoptotic pathway with coincident reduction in HRV infectious titre (Fig. 4a). However the addition of a specific caspase 8 inhibitor 1 hour after apoptosis induction led to an almost normal level of infectious virus titre (Fig. 4a); this is in agreement with a previous report^33^, suggesting that some caspase activation is required for effective virus replication. These data also suggest that the early pro-apoptotic effects of caspase 8 activation are not deleterious to virus replication. Interestingly, there was an increase in viral protein expression (VP2) with ActoD treatment (Fig. S1), suggesting that the induction of extrinsic apoptosis inhibits viral assembly or release but doesn’t inhibit viral protein synthesis. This indicates that the decrease in virus titre was not due to any decrease in viral protein synthesis and further suggests that a decrease in viral titre is most likely due to the apoptotic effects of ActoD.

Further evidence of HRV-induced changes to apoptotic pathways is observed in the flow cytometry results (Fig. 4b and c), which demonstrate a clear shift in the apoptotic state of cells. The treatment of cells with ActoD led to an increase in early and late apoptotic cells compared to the control, as expected. In contrast the proportion of late apoptotic cells is decreased with HRV16 infection of ActoD cells (Fig. 4b and c), with a coincident increase in the proportion of cells in early apoptosis as compared to ActoD alone (Fig. 4c). Together this suggests that the apoptotic pathways induced by ActoD did not to proceed into a late apoptotic state when cells are infected with HRV16 for 12 hours, probably due to viral restriction of apoptosis. Furthermore, the amount of caspase 3 activation in cells infected with HRV16 and treated with ActoD was increased with Rupintrivir treatment (Fig. 2e), further strengthening the proposed role of the 3C protease in antagonising apoptosis pathways.

As induction of apoptosis reduces production of infectious virus particles, it can be deduced that subsequent cleavage of apoptotic factors by HRV would facilitate viral production; indeed *Picornaviridae* proteases have previously been implicated in cleaving MDA-5 and RIG-I^13,34^ both of which have apoptosis inducing effects through caspase activation and recruitment domains. An essential adaptor protein, IPS-1, that facilitates the association of RIG-I and MDA-5 complexes, is also cleaved by *Picornaviridae* 3C and 2A proteases^13,34,35^, suggesting a mechanism by which induction of a host immune response is suppressed by Picornavirus. The TLR-3 and RIPK1 adaptor protein TRIF is also cleaved in coxsackievirus B infection^35^, further identifying the importance of disrupting these apoptotic pathways for Picornavirus infection. The common modulation of these pro-apoptotic proteins supports the data presented herein, that the cleavage of RIPK1 by HRV is imperative to changing host responses and establishing viral infection. Furthermore, inhibition of apoptosis may be a common attribute of picornaviruses; in addition to the examples above, procaspase 9 is cleaved by poliovirus *in vitro*^36^.

**Figure 5:**
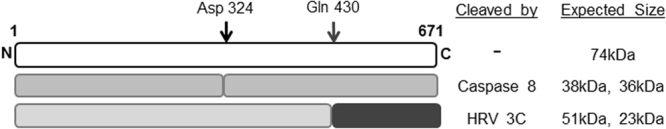
Proposed model of RIPK1 cleavage by HRV 3C within the caspase 8 generated, pro-apoptotic RIPK1 fragment. RIPK1 is cleaved by caspase 8 at Aspartic acid 324, inducing formation of a c-terminal, pro-apoptotic RIPK1 fragment early in apoptosis (19). HRV16 3C cleavage is predicted to occur at Glutamine 430, within the caspase 8 generated cleavage product and may generate an anti-apoptotic RIPK1 cleavage product.

Modulation of RIPK1 specifically may be a mechanism by which HRV modifies apoptotic pathways, conferring a cellular environment advantageous for viral replication. This hypothesis is supported by *in silico* sequence analysis of RIPK1 that showed a predicted HRV 3C cleavage site at Glutamine 430^37^, downstream of the caspase 8 cleavage site (Aspartic acid 324)^15^, potentially generating a ~25 kDa c-terminal RIPK1 product and a ~50 kDa n-terminal RIPK1 product, similar to that observed in this study. Throughout this study, two RIPK1 antibodies were used; the n-terminal RIPK1 antibody detected full length RIPK1 and a 50 kDa cleavage product (Fig. 2d and f) and the c-term antibody used detected full length RIPK1 and a 23 kDa cleavage product (Figs 1b,c, 2a and 3a). As described in this model (Fig. 5), the cleavage of RIPK1 at Glutamine 430 may disrupt the pro-apoptotic activity of the caspase 8 generated c-terminal RIPK1 fragment; supported by our data that the cell death profile (Fig. 4b) of ActoD treated cells differed between HRV16 infected and uninfected cells. Additional mechanistic studies are required to confirm the link between RIPK1 cleavage and the alteration of cell death pathways.

Together, our data suggest a scenario wherein initial induction of extrinsic apoptosis and cleavage of RIPK1 by caspase 8 in response to HRV infection may be prevented from proceeding to full apoptosis by cleavage of the pro-apoptotic RIPK1 fragment by HRV 3C protease. The 3C cleavage site and whether RIPK1 is a common substrate of all HRV subtypes remains to be elucidated.

## Methods

### Reagents

Actinomycin D (ActoD) (Sigma-Aldrich) was used at a concentration of 5 µg/mL to induce apoptosis. The pan-caspase inhibitor z.vad.FMK (BD Pharmigen) was used at concentration of 20 µM. Caspase inhibitors were purchased from Calbiochem and used at 4 µM (caspase 8 inhibitor) or as indicated. DMSO only controls were used to account for any cellular or viral alterations induced by the drug vehicle. The HRV 3C protease inhibitor, Rupintrivir (Santa Cruz, SC-208317) was added to infected cells at 1 µM as indicated. Anti-RIPK1 (ThermoScientific, 1860909) or anti-RIPK1 n-terminal (Cell signalling 3493) antibodies were used to detect full length RIPK1 and its cleavage products. Antibodies to caspase 3 (9662), Poly(A)-binding protein (PABP, 4992) and tubulin (2148) were purchased from Cell Signalling Technologies. Antibodies to poly-histidine tag (11922416001) Green Fluorescent protein (GFP, 11814460001) were purchased from Roche. Viral protein expression was detected with anti-VP2 antibody (QED Biosciences, 18758). Rabbit polyclonal antibody to eIF4G (sc11373) was purchased from Santa Cruz.

### Cells and virus

A549 cells (ECACC: 86012804) were used for all transfections and maintained in Dulbecco’s modified eagle medium (DMEM) supplemented with 10% fetal bovine serum (FBS) and Penicillin, Streptomycin and Neomycin (PSN, Gibco).

Ohio-HeLa (O-HeLa) cells (ECACC: 84121901) were used for infections throughout this study and maintained in DMEM supplemented with 10% FBS and PSN. All apoptosis or viral replication experiments were conducted in DMEM supplemented with 2% FBS and PSN. HRV serotype 16 was used for all experiments. HRV16 was cultured in O-HeLa cells as previously described^38^. Subconfluent monolayers of O-HeLa cells were infected with HRV16 at a multiplicity of infection (M.O.I) of 3 by absorption for 1 hour with occasional rocking, then removal of viral inoculum and replacement with DMEM supplemented with 2% FBS and PSN. Cells were treated with ActoD at 3 hours post infection (h.p.i) for 6 hours, z.vad.FMK at 4 h.p.i for 5 hours, caspase 8 inhibitor at 4 h.p.i for 5 hours or Rupintrivir at 9 h.p.i. for 7 hours. At indicated timepoints, cells were washed once with phosphate buffered saline (PBS; 137 mM NaCl, 2.7 mM KCl, 10 mM Na_2_HPO_4_, 1.7 mM KH_2_PO_4_, H_2_O (pH 7.4)) and incubated in RIPA buffer (150 mM NaCl, 1% triton-X, 0.5% sodium deoxycholate, 0.1% SDS, 50 mM Tris-HCl with phosphatase and protease inhibitors (Roche)) for 30 minutes on ice. Cell lysates were collected and centrifuged at 12,000 rpm for 10 minutes to remove cell debris. Supernatant was collected and Laemmli buffer was added before heating at 90 °C for 5 minutes as previously described^38^. Cell lysates were processed for Western blot analysis as described below.

### *In vitro* Protease Assay

Sub-confluent O-HeLa cells were treated with combinations of ActoD, z.vad.FMK or caspase 8 inhibitor (1 hour post ActoD treatment) or left untreated as indicated, and incubated for a total of 6 hours. Cells were lysed and proteins collected in RIPA buffer (without inhibitors) as described above. Lysates were incubated with 4U of recombinant HRV14 3C protease for 6 hours at 30 °C. The reaction was stopped with heating in Laemmli buffer for 5 minutes at 90 °C. Samples were then processed for Western Blot analysis as described below.

### Transfection

Previously described^38^ constructs encoding GFP tagged active HRV 3C protease (GFP-3C) or the inactive 3C protease (GFP-3Cinac) were transfected into a subconfluent monolayer of A549 cells with Lipofectamine 2000 reagent (Thermofisher) as per manufacturer’s instructions. At 16 hours post transfection, transfected cells or untransfected control cells were washed once with PBS, lysed in RIPA buffer as described above and processed for Western Blot as described below.

### Western Blot analysis

Cell lysates were subjected to SDS-polyacrylamide gel electrophoresis (SDS-PAGE) on 12.5% polyacrylamide gels and transferred to nitrocellulose membrane in Tris-Glycine-ethanol buffer (25 mM Tris Base, 192 mM Glycine, 20% Ethanol) for 90 minutes at 400mAmps. Transfer of proteins was confirmed with staining with Ponceau S Red (Sigma). Nonspecific binding sites were blocked with 4% skim milk or BSA in PBS. Blots were incubated overnight at 4 °C in primary antibody diluted in 1% skim milk or BSA in PBST (PBS with 0.1% Tween 20). After overnight incubation blots were washed in PBST and incubated in species specific secondary antibody conjugated to horseradish peroxidase diluted 1:5000 in 1% skim milk in PBST. Bound antibodies were detected with Enhanced Chemiluminiscence (ECL, Perkin Elmer) and exposure on Li-Cor Odyssey Fc Imager. Where appropriate, digital images were analysed using ImageJ to estimate protein levels relative to tubulin and values expressed as arbitrary units normalised to tubulin.

### Viral Replication kinetics

Sub-confluent O-HeLa cells were infected with HRV16 at M.O.I of 3 by absorption for 1 hour with occasional rocking, followed by replacement of inoculum with DMEM supplemented with 2% FBS and PSN. At 3 h.p.i cells were treated with ActoD at 5 µg/mL or remained untreated, as indicated. At 4 h.p.i either z.vad.FMK (20 µM) or caspase 8 inhibitor (4 µM) were added, as indicated. At indicated timepoints, cultures were frozen at −80 °C. Samples were thawed at room temperature and centrifuged to remove cellular debris, followed by titration as previously described^38^.

The viral titre was calculated as mean of three titres for each condition as determined by the Reed and Muench Calculator^39^. Viral titres are expressed as mean+/− standard error of the mean (SEM).

### Flow Cytometry Apoptosis Assay

O-HeLa cells were infected with HRV16 at M.O.I of 3 as described above or left untreated. At 3 h.p.i cells were treated with ActoD at 5 µg/mL or left untreated. Cells were collected and resuspended in Annexin V binding buffer (HEPES; 10 mM, NaCl; 140 mM, CaCl_2_; 2.5 mM) and stained with Annexin V-FITC conjugate (ThermoFisher: A13199) and Propidium Iodide (Pi) (BD: 556463) before flow cytometric analysis as per manufacturer’s instructions. BD LSRII cytometer and BD FACSDiva 8.0.1 were used for data acquisition.

### Statistical Analysis

Graphpad Prism 6 was used to determine statistical significance between relative estimated protein levels normalised to tubulin. Unless indicated otherwise, experiments were repeated three times and statistical significance was determined with a 2-tailed unpaired t-test, assuming equal variance of standard deviation. Statistical significance was accepted at p < 0.05.

## Electronic supplementary material

Supplementary Information
