# Supplementary material for: Human Rhinovirus 3C protease cleaves RIPK1, concurrent with caspase 8 activation
Source: Sci Rep. 2018 Jan 25;8:1569. doi: 10.1038/s41598-018-19839-4 (PMC5785518; doi:10.1038/s41598-018-19839-4)
Supplement: Supplementary file 1 — Supplementary Information [file 41598_2018_19839_MOESM1_ESM.pdf]

Human Rhinovirus 3C protease cleaves RIPK1, concurrent with caspase 8 activation

Sarah N Croft, Erin J Walker[#], Reena Ghildyal*[#]

Figure S1

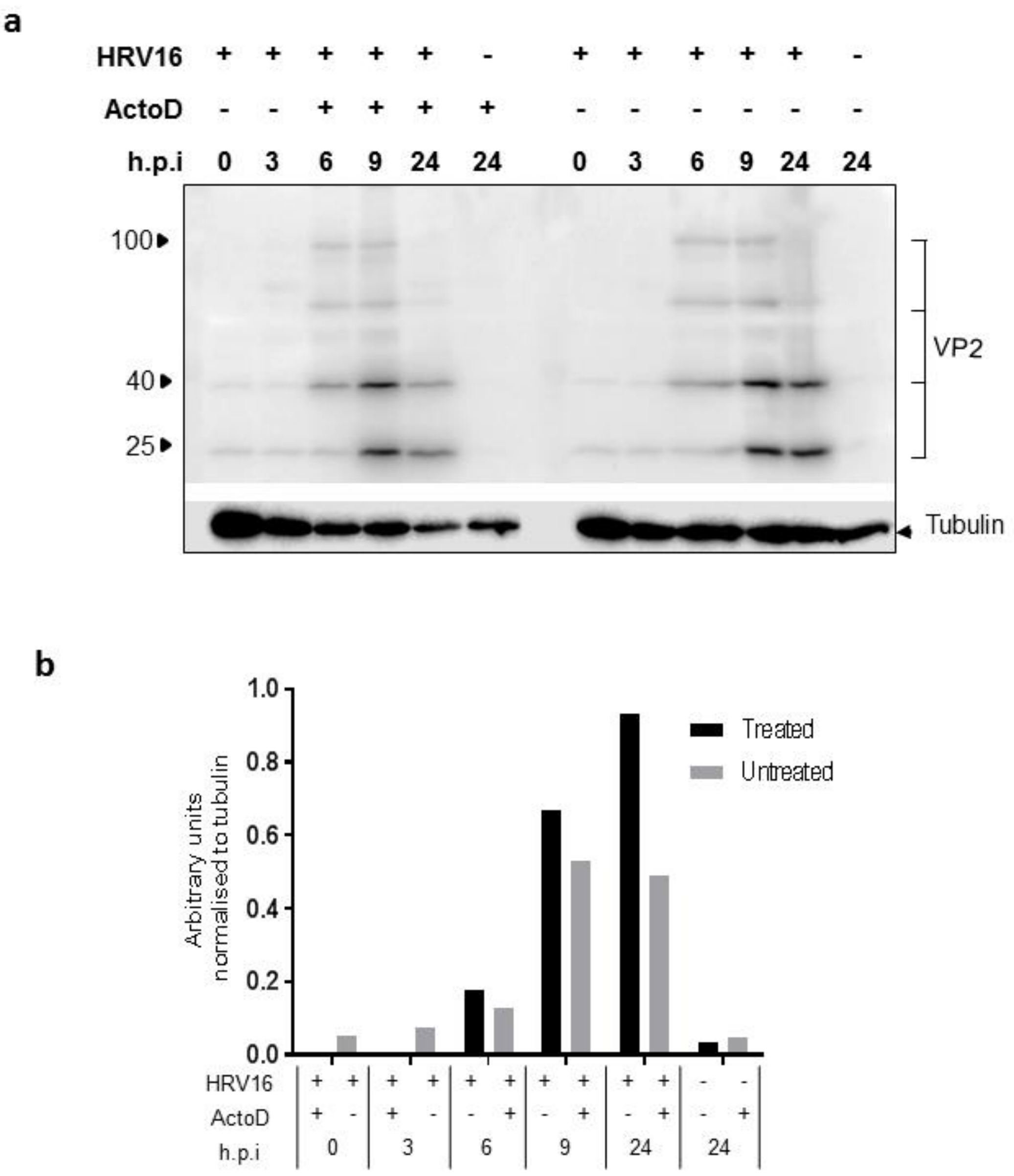
a
HRV16 + + + + + - + + + + + -
ActoD - - + + + + - - - - - -
h.p.i 0 3 6 9 24 24 0 3 6 9 24 24
100
40
25
VP2
Tubulin
b
Arbitrary units normalised to tubulin
1.0
0.8
0.6
0.4
0.2
0.0
Treated
Untreated
HRV16
ActoD
h.p.i
0
3
6
9
24
24

Figure S2

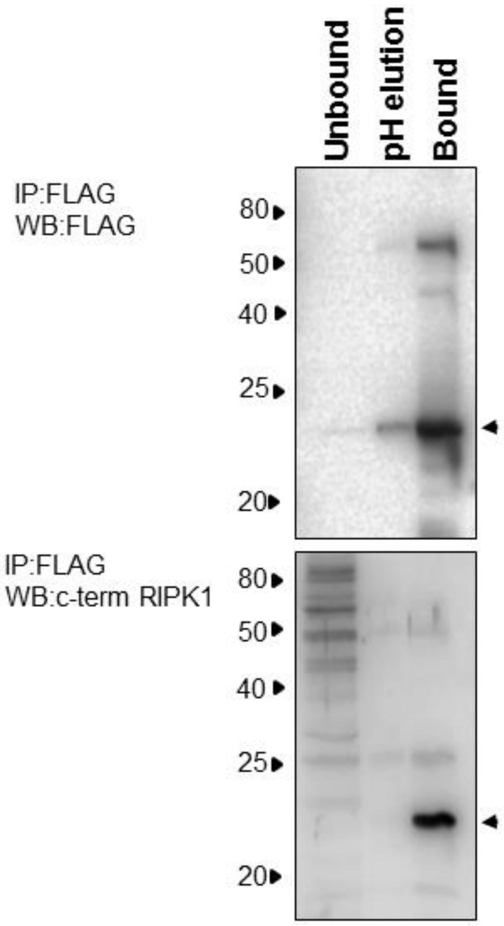

Unbound
pH elution
Bound
IP:FLAG
WB:FLAG
80
50
40
25
20
IP:FLAG
WB:c-term RIPK1
80
50
40
25
20

Figure S1. HRV16 capsid protein is increased with ActoD treatment. O-HeLa cells were either subject to infection with HRV16 at an M.O.I of 3 or left uninfected. At 3 h.p.i a subset of samples were treated with ActoD at 5µg/mL as indicated. Cells were then lysed at indicated times (h. p. i.) and proteins collected for western blot analysis.

a) Membrane was probed with primary anti-VP2 antibody, with anti-tubulin antibody included as a loading control. Antibodies are indicated on the right and molecular weight markers on the left of the blot.

b) Band intensities representative of the 35kDa VP2 detected in Figure S1a, were quantitated using ImageJ. Results were normalised to corresponding tubulin.

Figure S2. HRV 3C protease cleaves RIPK1 and produces a c-terminal fragment of ~25kDa. O-HeLa cells were transfected with a c-terminally FLAG tagged pcDNA3.1_hs_RIP1 construct with Lipofectamine 2000 as per manufacturer's instructions. After 16 hours of transfection, cells were collected and lysed. Cell lysates were incubated for 6 hours with the HRV14 3C protease and processed for an anti-FLAG immunoprecipitation. Anti-FLAG M2 beads were used to capture FLAG tagged proteins and 3 fractions were collected; proteins not bound to anti-FLAG antibodies (unbound), proteins eluted from the anti-FLAG antibodies with a low-pH elution buffer (pH elution), and those bound to anti-FLAG antibodies. Proteins were processed for western blot analysis.

Membrane was probed with anti-FLAG M2 antibody to confirm capture of FLAG tagged protein. An antibody specific to the c-terminal of RIPK1 (amino acids 611-625) was used to confirm the fragment of RIPK1. Antibodies are indicated on the left

of the blot and molecular weight markers on the left of the blot. Arrowhead denotes detected protein of interest.
